# The Binding of Fibroblast Growth Factor 23 (FGF23) to FGF Receptor‐αKlotho Complex Increases Sinoatrial Electrical Activity

**DOI:** 10.1111/apha.70293

**Published:** 2026-09-01

**Authors:** Giorgia Bertoli, Patrizia Benzoni, Serena Canzolino, David Molla, Raffaella Milanesi, Chiara Piantoni, Makoto Kuro‐o, Carmen Delgado, Gema Ruiz‐Hurtado, Mirko Baruscotti, Annalisa Bucchi

**Affiliations:** ^1^ The Cell Physiology MiLab, Department of Biosciences Università Degli Studi di Milano Milan Italy; ^2^ The Leon Charney Division of Cardiology New York University Grossmann School of Medicine New York New York USA; ^3^ Department of Veterinary Medicine and Animal Sciences Università degli Studi di Milano Milan Italy; ^4^ Institute of Cardiovascular Physiology and Pathophysiology Walter Brendel Center for Experimental Medicine, Biomedical Center (BMC), LMU München Munich Germany; ^5^ Division of Mineral Metabolism, Center for Molecular Medicine Jichi Medical University Tochigi Japan; ^6^ Instituto de Investigaciones Biomédicas Sols‐Morreale (CSIC‐UAM) Madrid Spain; ^7^ Centro de Investigación Biomédica en Red Enfermedades Cardiovasculares (CIBERCV) Madrid Spain; ^8^ Cardiorenal Translational Laboratory, Imas12 Research Institute Hospital Universitario 12 de Octubre Madrid Spain; ^9^ RICORS2040‐Renal, ISCIII Madrid Spain; ^10^ Department of Physiology, School of Medicine Universidad Autónoma de Madrid Madrid Spain

**Keywords:** α‐Klotho, FGF23, pacemaker current, sinoatrial node

## Abstract

**Aim:**

This study aims to investigate whether the Fibroblast Growth Factor 23 (FGF23) modulates the electrical activity of sinoatrial (SAN) cells. The canonical function of FGF23 is to regulate body phosphorus and calcium homeostasis by activating the FGF1 receptors (FGFR1)/α‐Klotho complex in the kidney and parathyroid glands. High levels of FGF23 can induce cardiac arrhythmias by affecting cardiomyocyte's function in an α‐Klotho independent manner. Although SAN cells are not traditionally considered targets of FGF23, the presence of α‐Klotho in pacemaker and not in ventricular cells has raised this possibility.

**Methods:**

The effect of FGF23 was evaluated by patch‐clamp experiments on mouse SAN and on human‐induced pluripotent stem cells‐derived pacemaker‐like cardiomyocytes (hiPSC‐derived pCMs).

**Results:**

Our data reveal that mouse SAN cells express both membrane α‐Klotho and FGF23 receptors (FGFR) and that 48 h tissue incubation with FGF23 (10 ng/mL) increases the spontaneous action potential (AP) frequency of these cells through an increase in the funny *I*_f_ current. Patch‐clamp experiments carried out using the pan‐FGFR inhibitor, PD173074, and SAN cells isolated from α‐Klotho hypomorphic mice suggested that FGF23 effects are mediated by the activation of the FGFR‐α‐Klotho complex. FGFRs expression data and FGF23‐induced electrical modification were further confirmed in hiPSC‐derived pCMs. Indeed, 48 h incubation of these cells with FGF23 increases both the AP frequency, in a dose‐dependent manner, and the *I*_f_ current.

**Conclusions:**

This study represents the first evidence that FGF23 directly regulates the SAN electrical activity.

## 1 Introduction

The Fibroblast Growth Factor 23 (FGF23) is a hormone mainly produced by osteocytes and osteoblasts, and its canonical effect is to lower serum phosphate levels by decreasing kidney phosphate reabsorption, 1,25‐dihydroxyvitamin D synthesis, and parathyroid hormone secretion by the parathyroid gland [1]. These effects are mediated by the activation of the ERK pathway [2] which is initiated by the binding of FGF23 to its receptor complex formed by the FGF Receptor 1 (FGFR1) and by the cofactor α‐Klotho. With particular regard to the kidney, this signaling cascade is considered the canonical pathway of FGF23. α‐Klotho was identified in 1997 by Kuro‐o et al. [3] as an aging‐suppressor factor. Two different biological active forms of α‐Klotho have been detected in humans and mice: a full‐length transmembrane form and a soluble form that originates from the cleavage of the extracellular domain of transmembrane α‐Klotho [4]. While the full‐length transmembrane form associates with FGFR1 and increases the affinity of the receptor for FGF23, the soluble form acts as an endocrine hormone that inhibits the pathways linked to aging and thus counteracts age‐related pathologies [4, 5, 6, 7].

A variety of studies have shown that high levels of FGF23, as observed for example in chronic kidney disease (CDK), are associated with cardiovascular diseases such as heart failure and arrhythmias [8]. Additionally, in vivo and in vitro data have demonstrated the ability of FGF23 to promote pathological cardiac remodeling such as ventricular hypertrophy, myocardial fibrosis, and ventricular Ca^2+^‐handling and ion channel dysfunctions [9]. Recent studies demonstrated that FGF23 can also act as a paracrine factor since it can be produced by cardiac myocytes and its release is enhanced during cardiac remodeling and heart failure, regardless of preserved or reduced renal function [10]. Ventricular myocytes do not express α‐Klotho and therefore the pro‐hypertrophic effects of FGF23 are mediated by a noncanonical pathway involving the activation of the FGF Receptor 4 (FGFR4) and the downstream calcineurin/NFAT and calmodulin kinase type II (CaMKII)‐dependent pathways ultimately causing left ventricular hypertrophy [11, 12]. Unlike ventricular myocytes, sinoatrial nodal cells express α‐Klotho and homozygous mutant mice with a defect in α‐Klotho gene expression (*kl/kl*) display intrinsic bradycardia and die of sinus arrest when exposed to restraining stress [13]. In addition, a recent article has identified an association between α‐Klotho mutations and a decrease in heart rate (HR) [14]. A significant role of α‐Klotho in sinoatrial node (SAN) function must then be presumed. In this study, we therefore investigated whether FGF23 modulates the pacemaker properties of murine SAN cells and of human‐induced pluripotent stem cells (iPSC)‐derived pacemaker cardiomyocytes (pCMs). In addition, we tested whether the FGF23 actions depend on the presence of Klotho.

## 2 Results

### 2.1 Murine SAN Cells Express Canonical FGF23 Receptors

**FIGURE 1:**
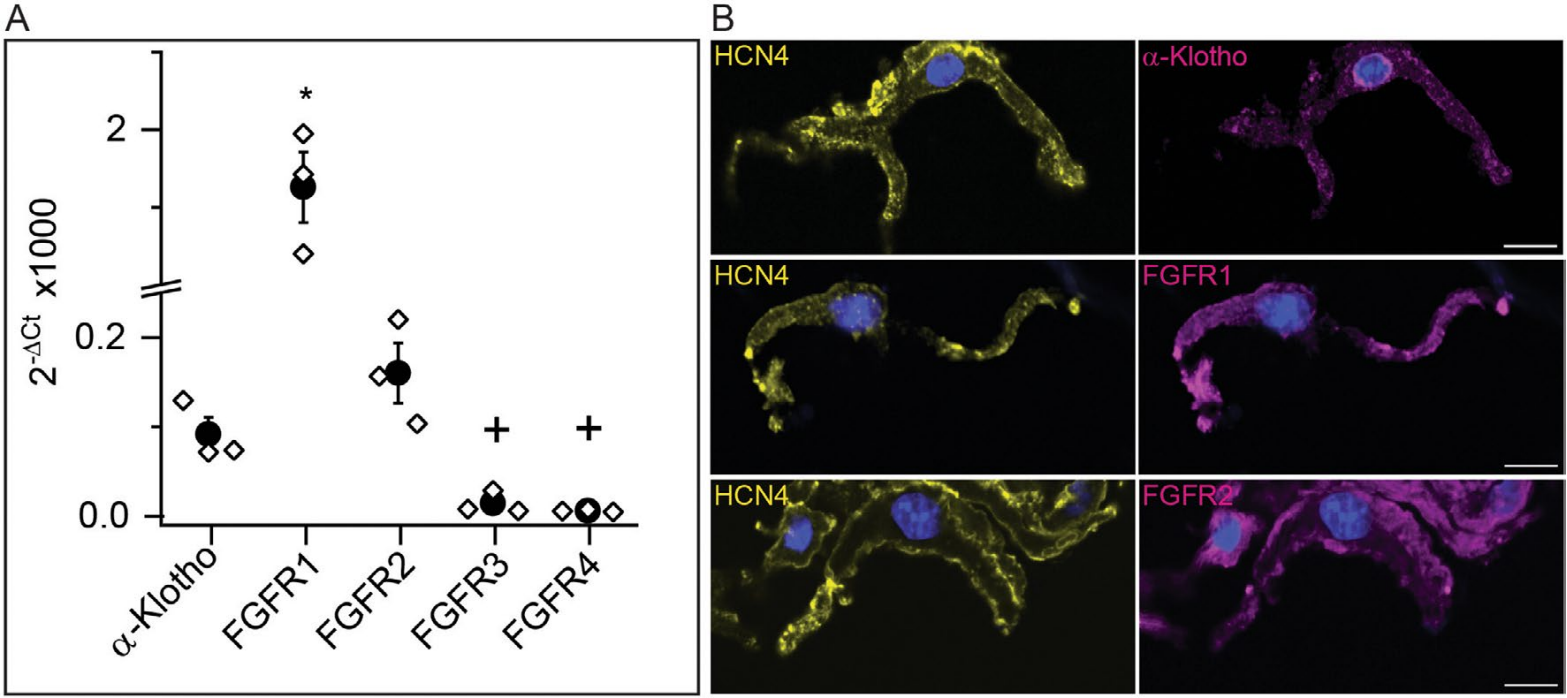
SAN cells express FGFRs and α‐Klotho. (A) Mean (black circle) ± SEM and single (open diamonds) data of RT‐qPCR analysis of α‐Klotho and FGFR1‐4 mRNAs in SAN tissue (*n* = 3). α‐Actin was used for normalization. *p < 0.005 versus α‐Klotho, FGFR2, 3 and 4; +*p* < 0.005 versus α‐Klotho, FGFR1, and 2 (One‐way ANOVA, followed by Bonferroni test on ln transformed data). (B) Representative SAN cells staining for HCN4 (yellow), α‐Klotho (magenta, top), FGFR1 (magenta, middle), and FGFR2 (magenta, bottom). Nuclei were stained with Hoechst (blue). Scale bar 10 μm.

RT‐qPCR analysis was used to investigate in murine SAN tissue the relative abundance of mRNAs encoding for different members of the FGFR family (FGFR1‐4) and for the membrane form of the cofactor α‐Klotho (Figure 1A). FGFR1 signal was the most abundant followed by FGFR2 and α‐Klotho; FGFR3 and FGFR4 were also expressed, albeit at negligible levels close to the limit of detection. The presence of α‐Klotho, FGFR1, and FGFR2 proteins in SAN cells was confirmed by immunostaining. Images in Figure 1B and Figure S1A demonstrate that SAN cells, identified both by the typical shape and by the presence of the HCN4 pacemaker channel [15], a specific SAN cell marker, express α‐Klotho, FGFR1, and FGFR2.

### 2.2 In Vitro Prolonged (48 h) SAN Tissue Exposure to FGF23 Increases the Spontaneous Activity of Pacemaker Cells Through the Activation of FGFR

**FIGURE 2:**
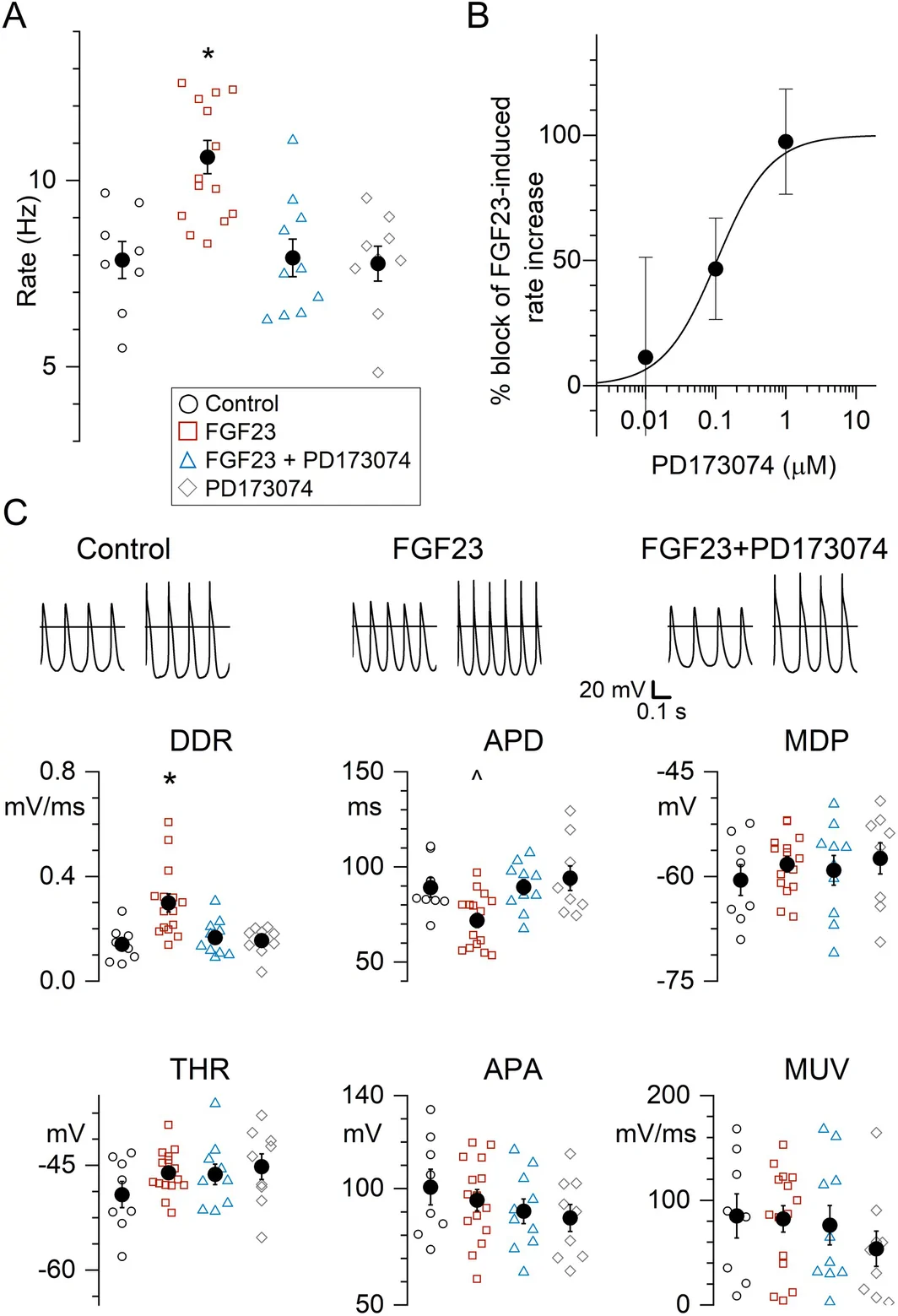
Prolonged (48 h) exposure to FGF23 10 ng/mL increases the AP rate of SAN cells through FGFR activation. (A) Mean (filled circles) ± SEM and single AP rate data obtained after 48 h SAN incubation with vehicle (control, open circles), FGF23 10 ng/mL (open squares), FGF23 10 ng/mL + PD173074 1 μM (open triangles), and PD173074 1 μM (open diamonds). *p < 0.05 versus control, FGF23 10 ng/mL + PD173074 1 μM, and PD173074 1 μM (one‐way ANOVA, followed by Bonferroni). (B) Dose–response inhibition of the FG23 10 ng/mL‐induced rate increase by PD173074. Mean AP rate (± SEM) obtained after 48 h tissue incubation with FGF23 10 ng/mL + PD173074 0.01 μM, FGF23 10 ng/mL + PD173074 0.1 μM, and FGF23 10 ng/mL + PD173074 1 μM were 9.99 ± 0.96 Hz *n* = 6 cells/4 mice, 9.14 ± 0.49 Hz *n* = 16 cells/9 mice, and 7.92 ± 0.50 Hz *n* = 10 cells/4 mice, respectively. Each point represents the % mean of the FG23 10 ng/mL‐induced rate increase inhibition by PD173074. The Hill fitting yielded the following values: *K* = 103.7 nM, and *h* = 1.1 (*y*_max_ was fixed at 100). (C) Sample AP traces and AP parameters obtained in control, FGF23 10 ng/mL, FGF23 10 ng/mL + PD173074 1 μM, and PD173074 1 μM (symbols as in A). *p < 0.05 versus vehicle (control), FGF23 10 ng/mL + PD173074 1 μM, and PD173074 1 μM; ^*p* < 0.05 versus FGF23 + PD173074 and PD173074 (one‐way ANOVA, followed by Bonferroni test).

Given the robust expression of FGFR1, FGFR2, and α‐Klotho, we next evaluated whether FGF23 could modulate the activity of SAN cells. To this aim, we exposed SAN cells either to vehicle (control) or to FGF23 (10 ng/mL) for various durations (74–120 s; 30–60 min; 48 h) and spontaneous APs were recorded. In the first two sets of experiments (74–120 s and 30–60 min), the exposure to FGF23 was performed on isolated cells obtained just after SAN dissection. For long exposure experiments, the whole SAN tissue was incubated for 48 h with vehicle or FGF23. At the end of the incubation period, cells were isolated and patch‐clamp experiments were performed. We chose to assess the effect of different exposure times to FGF23 stimulation since previous evidence indicated that changes in electrical/mechanical properties and/or morphology of cardiac cells required durations ranging from a few minutes to days [11, 16, 17, 18]. Data in Figure 2A and Table 1 show that after 48 h of FGF23 (10 ng/mL) incubation, the spontaneous rate of SAN cells was significantly increased by 35.1%. This increase was fully reversed when the SAN tissue was incubated with both FGF23 and the FGFR blocker PD173074 (1 μM). A more complete description of this inhibition is presented in Figure 2B where the dose–response of the PD173074 inhibition of the FGF23 (10 ng/mL)‐induced rate increase is shown. This inhibition, together with the evidence that the incubation with the blocker (1 μM) alone does not affect the spontaneous activity of SAN cells (Figure 2A, Table 1), clearly indicates that the increase of the AP frequency specifically depends on the FGFR stimulation and on the associated signaling cascade. Notably, when shorter FGF23 exposures (74–120 s, Figure S2A or 30–60 min, Figure S2C) were tested on AP rate, no effect could be detected, demonstrating a lack of efficacy in acute condition.

**TABLE 1:** Mean ± SEM values of AP parameters obtained in SAN cells treated for 48 h with vehicle (control), FGF23 10 ng/mL, FGF23 10 ng/mL + PD173074 1 μM, and PD173074 1 μM.

	Control (*n* = 8 cells/5 mice)	FGF23 (*n* = 15 cells/10 mice)	FGF23 + PD173074 (*n* = 10 cells/4 mice)	PD173074 (*n* = 9 cells/5 mice)
Rate (Hz)	7.87 ± 0.50	10.63 ± 0.45*	7.92 ± 0.50 Hz	7.77 ± 0.47
DDR (mV/ms)	0.14 ± 0.02	0.30 ± 0.03*	0.17 ± 0.02	0.16 ± 0.02
APD (ms)	89.2 ± 5.1	71.9 ± 3.7^†^	89.4 ± 4.0	94.1 ± 6.5
MDP (mV)	−60.5 ± 2.2	−58.3 ± 1.1	−59.1 ± 2.1	−57.4 ± 2.2
THR (mV)	−49.2 ± 1.9	−46.1 ± 0.8	−46.3 ± 1.5	−45.2 ± 1.8
APA (mV)	100.7 ± 7.7	95.0 ± 4.6	90.3 ± 5.3	87.4 ± 5.8
MUV (mV/ms)	85.1 ± 21.1	82.1 ± 12.6	76.2 ± 18.8	53.7 ± 16.9

Abbreviations: APA, action potential amplitude; APD, action potential duration; DDR, diastolic depolarization rate; MDP, maximum diastolic potential; MUV, maximum upstroke velocity; THR, threshold.

* *p* < 0.05 versus vehicle (control), FGF23 + PD173074 and PD173074.

^†^ *p* < 0.05 versus FGF23 + PD173074 and PD173074 (one‐way ANOVA, followed by Bonferroni test).

To define the mechanisms responsible for the rate increase induced by FGF23 in the chronic setting, the AP parameters were analyzed. In agreement with data previously reported [19, 20], mouse APs exhibited a great waveform heterogeneity (Figure S3).

Two different representative AP traces recorded for each condition (control, FGF23, and FGF23 + PD173074) after 48 h‐treatment are shown in Figure 2C, top. AP parameters were calculated for each experimental condition and data are shown in Figure 2C, middle and bottom, and Table 1. Statistical analysis indicates that, when compared to control FGF23 increased the diastolic depolarization rate (DDR). No changes in the maximum diastolic potential (MDP), threshold (THR), action potential amplitude (APA), and maximum upstroke velocity (MUV) were observed. Since a mechanism based on an increase of the *I*_f_ current is coherent with these observations [21], we next tested the effects of FGF23 on this current.

### 2.3 In Vitro Prolonged (48 h) SAN Tissue Exposure to FGF23 Increases the *I*_f_ Current Through the Activation of the FGFR

**FIGURE 3:**
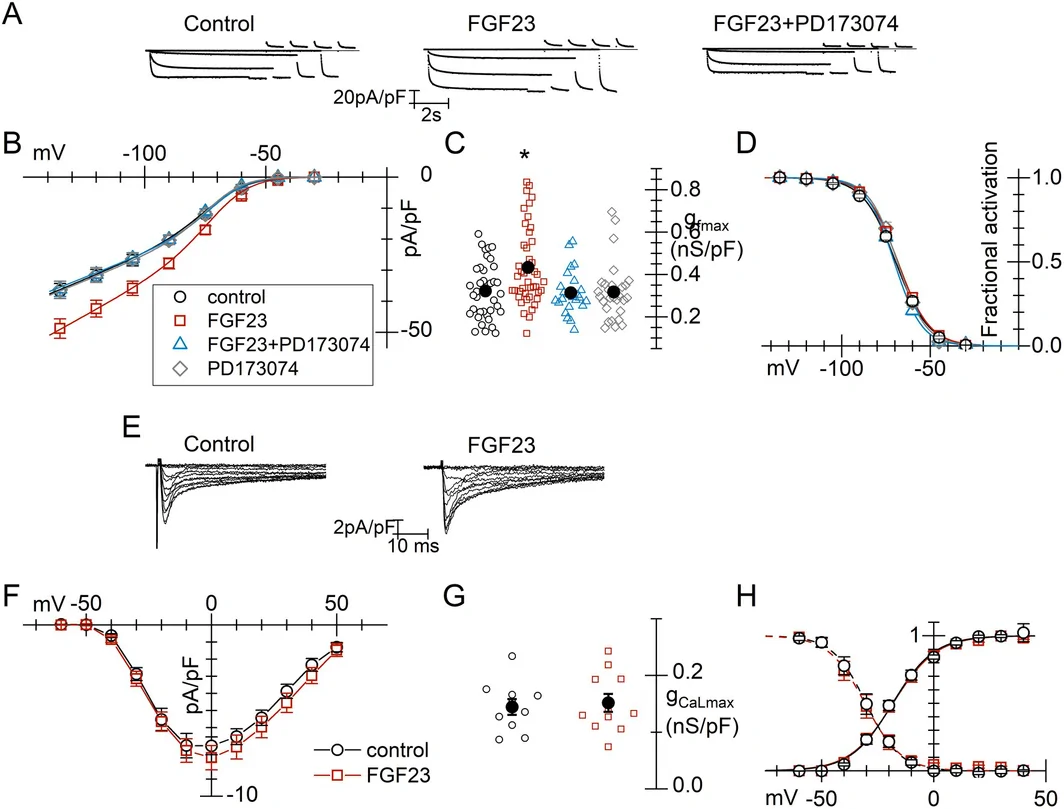
Prolonged (48 h) exposure to FGF23 10 ng/mL increases the *I*_f_ current through FGFR activation. (A) Representative *I*_f_ current traces recorded after 48 h of incubation with vehicle (control), FGF23 10 ng/mL, and FGF23 10 ng/mL + PD173074 1 μM. Currents densities obtained at −30, −60, −90 and −120 mV followed by a step to −125 mV and to 10 mV are shown (hp = −30 mV). (B) Mean steady‐state I/V curves obtained from cells treated for 48 h with vehicle (control, open circles; *n* = 38 cells/11 mice), FGF23 10 ng/mL (open squares, *n* = 44 cells/11 mice), FGF23 10 ng/mL + PD173074 1 μM (open triangles, *n* = 25 cells/6 mice), and PD173074 1 μM (open diamonds, *n* = 29 cells/6 mice). (C) Mean (black circles) ± SEM and single values of *g*_fmax_ (symbols as in B). *p < 0.05 versus vehicle (control), FGF23 10 ng/mL + PD173074 1 μM, and PD173074 1 μM (Kruskal‐Wallis ANOVA, followed by Dunn's test). (D) Mean *I*_f_ activation curves (symbols as in B). (E) Sample *I*_CaL_ (nifedipine‐sensitive) current traces, (F) mean steady‐state *I*_CaL_ IV curves, (G) mean (black circles) ± SEM and single values of *g*_CaLmax_, and (H) mean activation (solid lines) and inactivation curves (dashed lines) of *I*_CaL_ obtained after 48 h of incubation with vehicle (control, open circles) and with FGF23 10 ng/mL (open squares).

To test whether an increase of the *I*_f_ current is the mechanism underlying the rate increase induced by the growth factor, we evaluated the whole‐cell *I*_f_ current properties in single cells isolated after 48 h of SAN incubation with vehicle (control), FGF23 (10 ng/mL), FGF23 (10 ng/mL) + PD173074 (1 μM), and PD173074 (1 μM). As shown by the representative *I*_f_ current traces (Figure 3A) and by steady‐state I/V distributions (Figure 3B), the FGF23 treatment increased the *I*_f_ current. To better quantify this increase, we evaluated the effect of FGF23 both on the maximal conductance and on the voltage dependence of activation. Mean maximal *I*_f_ conductance density values (*g*_fmax_, Figure 3C, and Table 2) confirmed an FGF23‐induced significant increase of this parameter. This increase was completely abolished by the concomitant presence of the receptor blocker PD173074 1 μM; PD173074 alone had no effect. The evaluation of the voltage dependence of the *I*_f_ activation in the four experimental conditions is shown in Figure 3D and Table 2; no significant difference of the half‐activation voltages (*V*_1/2_) was found among the four different conditions. In agreement with results on APs, we observed that short (90–150 s, Figure S2B) or intermediate (30–60 min, Figure S2D,E) exposures of SAN cells to FGF23 did not modify either the *I*_f_ conductance, or the activation curves.

**TABLE 2:** Mean ± SEM *I*_f_ and *I*_CaL_ properties obtained from SAN cardiomyocytes treated for 48 h with vehicle (control), FGF23 10 ng/mL, FGF23 10 ng/mL + PD173074 1 μM, and PD173074 1 μM.

*I*_f_	Control (*n* = 38 cells/11 mice)	FGF23 (*n* = 44 cells/11 mice)	FGF23 + PD173074 (*n* = 25 cells/6 mice)	PD173074 (*n* = 29 cells/6 mice)
*g*_fmax_ (ns/pF)	0.32 ± 0.02	0.43 ± 0.03*	0.31 ± 0.02	0.32 ± 0.02
*V*_1/2_ (mV)	−69.7 ± 0.9	−67.9 ± 0.9	−70.3 ± 1.0	−68.7 ± 1.3
*s* (mV)	8.6 ± 0.3	8.1 ± 0.3	7.3 ± 0.3	7.0 ± 0.3^†^
*I*_CaL_	Control	FGF23		
*g*_CaLmax_ (ns/pF)	0.14 ± 0.01 (*n* = 10 cells/4 mice)	0.15 ± 0.02 (*n* = 11 cells/7 mice)		
*V*_1/2_ activation (mV)	−18.5 ± 1.6	−18.0 ± 1.7		
*s* activation (mV)	9.0 ± 0.4	9.1 ± 0.5		
*V*_1/2_ inactivation (mV)	−30.2 ± 2.4 (*n* = 4 cells/3 mice)	−28.8 ± 2.5 (*n* = 5 cells/5 mice)		
*s* inactivation (mV)	7.1 ± 0.1	7.4 ± 0.3		

*Note:* *g*_max_, maximal conductance density; *V*_1/2_, half‐activation voltage; *s*, inverse‐slope factor.

* *p* < 0.05 versus vehicle (control), FGF2310ng/mL + PD173074 1 μM, and PD173074 1 μM.

^†^ *p* < 0.05 versus control (Kruskal–Wallis ANOVA, followed by Dunn's test).

Since the L‐Type calcium current (*I*_CaL_) also contributes to the auto‐rhythmicity of SAN cells [22, 23], we considered this current as an additional possible target of FGF23. Data shown in Figure 3E–H and Table 2, collected after 48 h of incubation with vehicle or FGF23 10 ng/mL, demonstrate that neither channel maximal conductance density (*g*_CaLmax_) nor the voltage‐dependence of activation and inactivation are modified by the growth factor.

### 2.4 α‐Klotho Is Necessary for FGF23‐Dependent *I*_f_ Modulation

**FIGURE 4:**
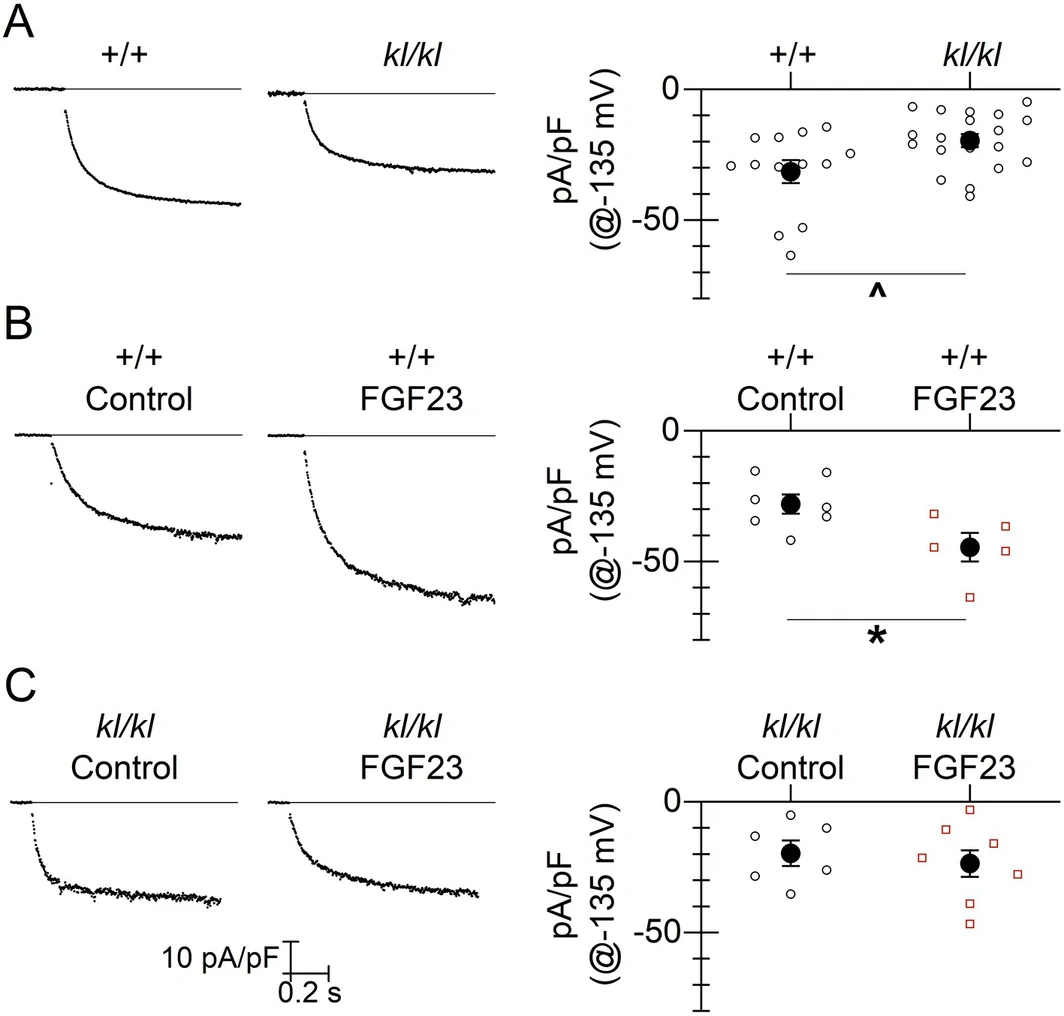
Prolonged (48 h)exposure to FGF23 10 ng/mL does not affect the *I*_f_ current density in *kl/kl* mice Representative *I*_f_ current traces recorded at −135 mV (hp −30 mV) and mean (black circles) ± SEM/single values (open symbols) of the *I*_f_ density at −135 mV recorded from freshly isolated SAN +/+ and *kl/kl* cells (A), from SAN +/+ cells incubated for 48 h with either vehicle or FGF23 10 ng/mL, (B), and from SAN *kl/kl* cells incubated for 48 h with either vehicle or FGF23 10 ng/mL (C). *p < 0.05 (Student's *t*‐test). ^*p* < 0.05 (Mann–Whitney test).

Previous results indicate a faster spontaneous AP rate of SAN cells after a long (48 h) exposure to FGF23, and this can be the consequence of an increase of the *I*_f_ maximal conductance. In addition, the ability of the FGFR blocker to inhibit the FGF23 action clearly points to a mechanism based on receptor activation and a downstream signaling cascade, ultimately leading to the *I*_f_ modulation. To better clarify the role of the co‐receptor α‐Klotho in these events, we evaluated the effect of FGF23 (10 ng/mL) on the *I*_f_ current in SAN cells isolated from mice hypomorphic for Klotho (*kl/kl*), and from their wild‐type (*+/+*) littermates. Since FGF23 increases the *g*_fmax_ value, we chose to measure the *I*_f_ current using a test step at −135 mV, a voltage that ensures the attainment of maximal conductance, and therefore represents a reliable readout of the FGF23 effect. *kl/kl* SAN cells, isolated immediately after tissue dissection, present with a reduced *I*_f_ current density compared to those obtained from +/+ SAN cells (−19.6 ± 2.5 pA/pF, *n* = 19 cells/7 mice versus −31.5 ± 4.4 pA/pF, *n* = 13 cells/5 mice; *p* < 0.05, Mann–Whitney test; Figure 4A).

These results are coherent with the evidence that mice with α‐Klotho deficiency display intrinsic bradycardia [13], and suggest that α‐Klotho also influences the *I*_f_ physiological levels. To evaluate if α‐Klotho is necessary for the FGF23‐mediated increase of the *I*_f_ current, SAN tissues dissected from +/+ and *kl/kl* mice were incubated for 48 h with either vehicle or FGF23 10 ng/mL, and after cell isolation, the *I*_f_ current was recorded. In agreement with the experiments of Figure 3A–C, FGF23 increased the *I*_f_ current density in +/+ cells (control: −28.0 ± 3.7 pA/pF, *n* = 7 cells/2 mice; FGF23: −44.5 ± 5.5 pA/pF, *n* = 5 cells/2 mice, *p* < 0.05 Student *t*‐test, Figure 4B), but did not affect *kl/kl* SAN cells (control: −19.7 ± 4.9 pA/pF, *n* = 6 cells/4 mice; FGF23: −23.6 ± 5.1 pA/pF, *n* = 8 cells/3 mice; *p* > 0.05 Student *t*‐test, Figure 4C). These data indicate that the FGF23‐dependent increase of the SAN *I*_f_ current requires the activation of the FGFR‐α‐Klotho complex.

### 2.5 FGF23 Increases the Spontaneous Activity and the *I*_f_ Current in Human Pacemaker‐Like Cardiac Cells

**FIGURE 5:**
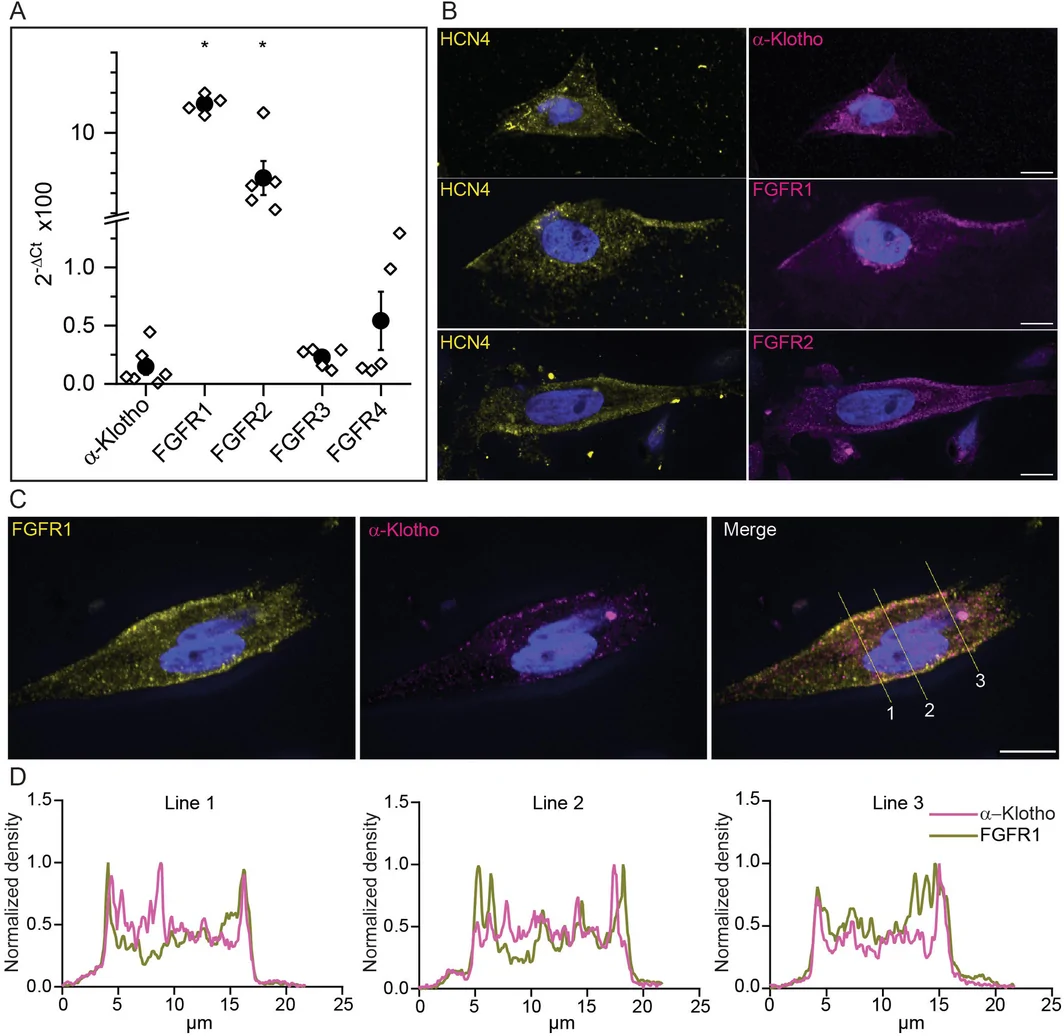
pCM express FGFRs and α‐Klotho. (A) Mean (black circle) ± SEM and single (open diamonds) data of RT‐qPCR analysis of α‐Klotho and FGFR1‐4 mRNAs in pCMs. Troponin T was used as reference gene. *p < 0.05 versus α‐Klotho, FGFR 3 and 4 (One way ANOVA, followed by Bonferroni test on ln transformed data). (B) Representative pCMs staining for HCN4 (yellow), α‐Klotho (magenta, top), FGFR1 (magenta, middle), and FGFR2 (magenta, bottom). (C) Representative immunofluorescence images showing the FGFR1 (yellow) and α‐Klotho (magenta) expression in the same pCM. (D) Normalized intensity profiles along three independent linear regions (lines 1–3 of merged image in panel C) for FGFR1 and Klotho. Nuclei were stained with Hoechst (blue). Scale bar 10 μm.

To further support the role of FGFR and α‐Klotho, we tested their presence and function in hiPSC‐derived pCMs. RT‐qPCR analysis showed, similarly to the mouse SAN data, a prevalent expression of FGFR1 and FGFR2 (Figure 5A; Figure S4). The presence of protein expression of α‐Klotho (Figure 5B top Figure S1B,C), FGFR1 (Figure 5B middle), and FGFR2 (Figure 5B bottom) was then confirmed by immunofluorescence experiments. Co‐immunolabeling of α‐Klotho and FGFR1 is presented in Figure 5C; co‐localization of the two proteins is suggested both by the merged co‐staining pattern and by the presence of overlapping peaks in the normalized linear intensity surface plots (Figure 5C,D).

**FIGURE 6:**
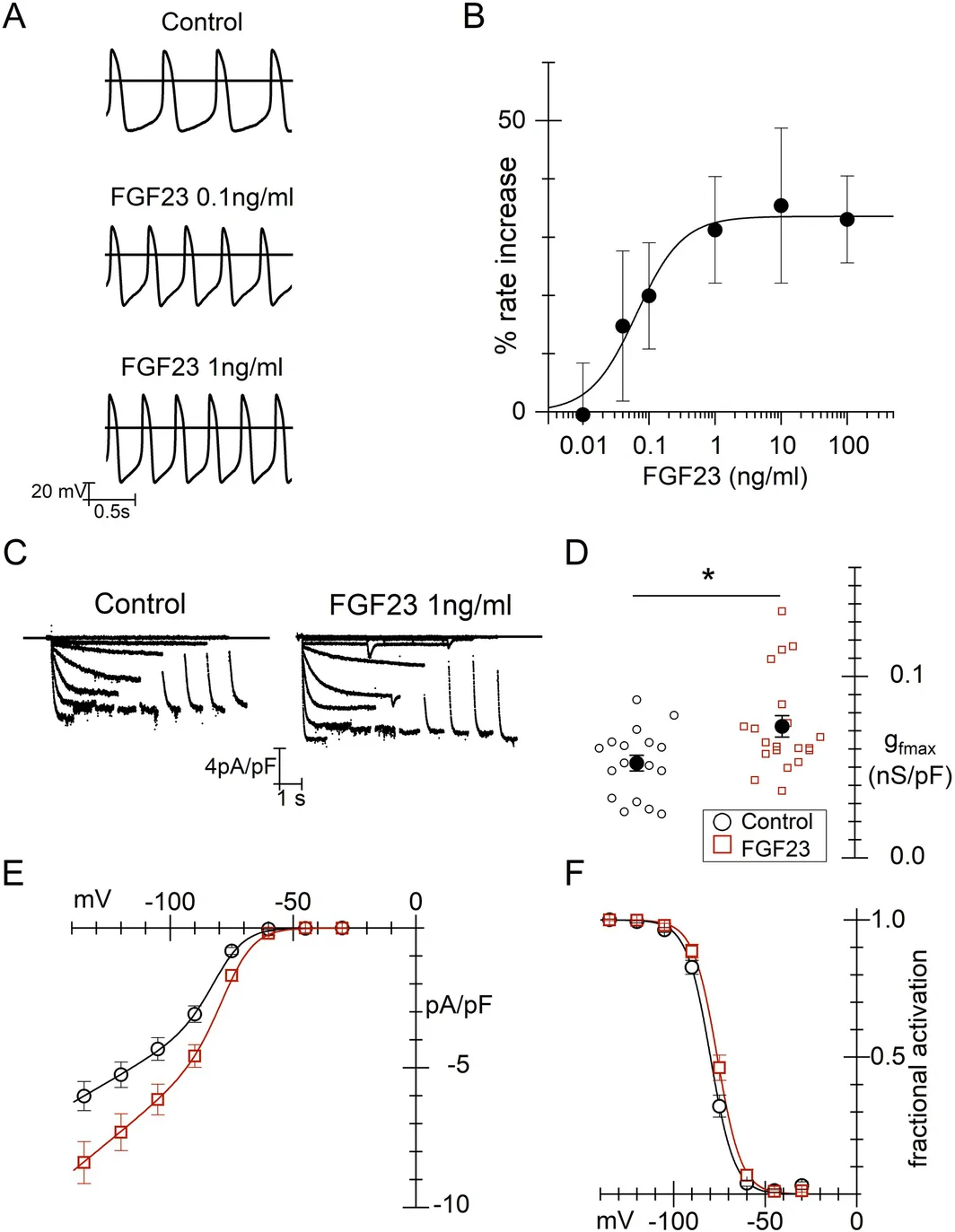
Prolonged (48 h) exposure to FGF23 increases the AP rate and *I*_f_ current of pCMs. (A) Representative AP traces recorded after 48 h of pCMs incubation with vehicle, FGF23 0.1 ng/mL, and FGF23 1 ng/mL. (B) Dose–response relationship of FGF23 rate increase effect. Each dot represents the mean ± SEM of 4–16 cells. (C) Representative *I*_f_ current traces recorded after 48 h of pCMs incubation with vehicle or FGF23 1 ng/mL. (D) Mean (black circles) ± SEM and single values of *g*_fmax_, (E) mean steady‐state IV relationships, and (F) mean *I*_f_ activation curves obtained after 48 h of pCMs incubation with vehicle (open circles, *n* = 18) or FGF23 1 ng/mL (open squares, *n* = 20). *p < 0.05 (Mann–Whitney test).

Next, we evaluated the effect of FGF23 on the APs and on the *I*_f_ current properties in this human substrate, by incubating pCMs for 48 h either with human FGF23 or with vehicle (control). After incubation, single cells were isolated, and patch‐clamp experiments were performed in the whole‐cell configuration. In Figure 6A representative sample AP traces, recorded after pCMs incubation with the vehicle and two different doses of FGF23 (0.1 and 1 ng/mL) are shown. FGF23 caused a dose‐dependent rate increase, and the Hill fitting of the experimental dose–response data points distribution yielded a half‐effect value (*k*) of 0.064 ng/mL, a maximal increase value (*y*_max_) of 33.6%, and a Hill coefficient (h) of 1.2 (Figure 6B).

**FIGURE 7:**
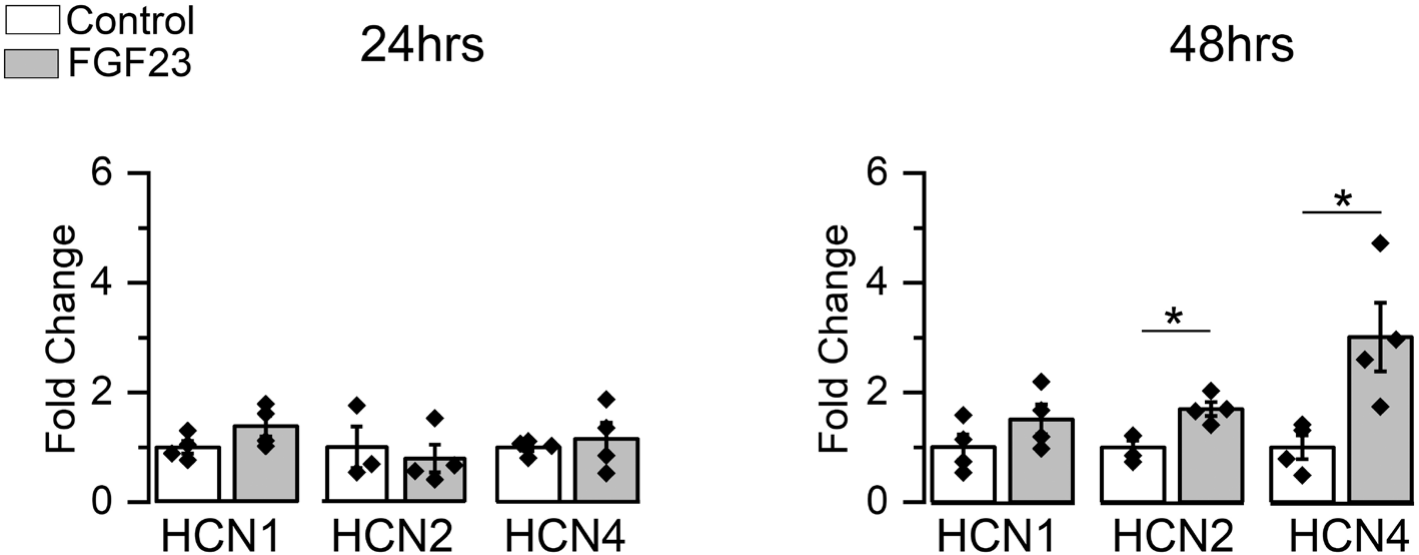
Prolonged (48 h) exposure to FGF23 increase HCN2 and HCN4 expression in pCMs. Fold‐change in mRNA levels of HCN1, HCN2, and HCN4 after 24 and 48 h of pCMs treatment with FGF23 1 ng/mL compared with control. RLP0 was used as reference gene. White bars represent vehicle‐treated cells; gray bars represent FGF23‐treated cells. Individual data points are shown as black diamonds. *p < 0.05, Student's *t*‐test.

FGF23 1 ng/mL increased rate by 31.1%, and the analysis of the AP parameters indicates that, as in mouse SAN cells, the growth factor increases the DDR (Table 3). In Figure 6C–F representative *I*_f_ current traces, *g*_fmax_ values, mean steady‐state IV curves, and mean activation curves of the *I*_f_ current in control and in the presence of FGF23 (1 ng/mL) are shown. As expected, 48 h treatment with FGF23 1 ng/mL increased maximal *g*_fmax_ (from 0.052 ± 0.004 nS/pF, *n* = 18 to 0.072 ± 0.006 nS/pF, *n* = 20; *p* < 0.05 Mann–Whitney test). Differently from mouse cells, in pCM cells, FGF23 was able to modulate the voltage dependence of activation of the *I*_f_ current by inducing a significant positive shift of 3.6 mV of the *V*_1/2_ value (from −79.8 ± 1.1 mV to −76.2 ± 1.3 mV, *p* < 0.05, Mann–Whitney test). The FGF23‐dependent increase in *g*_fmax_ strongly suggests an increased synthesis or decreased degradation of f‐channels. To test this hypothesis, we measured by RT‐qPCR the mRNA levels of f‐channel molecular components after 24 and 48 h pCM incubation with human FGF23 (1 ng/mL) or vehicle. As shown in Figure 7, FGF23 exposure does not alter the expression of HCN isoforms at 24 h; however, at 48 h both HCN2 and HCN4 show a significant upregulation. This indicates that the transcriptional response to FGF23 is a “late” (> 24 h) process and selectively targets specific HCN isoforms.

**TABLE 3:** Mean ± SEM values of AP parameters obtained in pCMs treated for 48 h with vehicle (control) and FGF23 1 ng/mL.

	Control (*n* = 20)	FGF23 (*n* = 16)
Rate (Hz)	2.22 ± 0.13	2.91 ± 0.20*
DDR (mV/ms)	0.09 ± 0.01	0.14 ± 0.02^†^
APD (mV)	172.7 ± 15.8	164.5 ± 17.4
MDP (mV)	−59.9 ± 1.3	−58.9 ± 1.3
THR (mV)	−29.5 ± 1.4	−31.0 ± 1.4
APA (mV)	92.9 ± 2.2	91.5 ± 2.5
MUV (mV/ms)	11.3 ± 0.8	12.1 ± 1.4

Abbreviations: APA, action potential amplitude; APD, action potential duration; DDR, diastolic depolarization rate; MDP, maximum diastolic potential; MUV, maximum upstroke velocity; THR, Threshold.

* *p* < 0.05, Student's *t*‐test.

^†^ *p* < 0.05, Mann–Whitney test.

## 3 Discussion

Our data indicate that mouse SAN cells express both the membrane form of α‐Klotho and the FGF Receptors (mainly FGFR1; Figure 1), and patch‐clamp experiments show that: (1) FGF23 increases the spontaneous AP frequency via an increase of the DDR slope (Figure 2A,C; Table 1); (2) FGF23 increases the *I*_f_ current conductance (Figure 3B,C; Table 2); (3) the effect of FGF23 on SAN electrical activity is specifically mediated by the binding to the FGFR since a pan‐FGFR blocker (PD173074) blunts this effect (Figures 2A,B and 3A–C, Tables 1 and 2); (4) the co‐factor α‐Klotho is a mandatory element of the transduction cascade since no effect on the *I*_f_ current is observed when SAN tissue isolated from *kl/kl* mice is treated with FGF23 (Figure 4C); (5) the action of FGF23 requires a prolonged exposure (48 h, Figures 2, 3, and Figure S2) and therefore it likely occurs via an increase of the f‐channels synthesis or a decrease of their degradation.

FGF23 can bind all of FGF receptors subtypes, however, when the FGFR1 specific co‐factor α‐Klotho is present, the affinity of the FGFR1‐α‐Klotho complex is much increased [5, 6]. The evidence presented in Figure 1 confirms the presence of both FGFR1 and α‐Klotho in pacemaker SAN cells, therefore it is the activation of this pathway that likely mediates the FGF23 effects on cardiac pacemaking.

Data obtained in mouse SAN were confirmed in the human pacemaker‐like cell model (pCM) which also expresses membrane α‐Klotho and FGFRs (Figure 5A,B). Indeed, the incubation with FGF23 for 48 h increased the AP frequency in a dose‐dependent manner (Figure 6A,B). Evaluation of the effect of FGF23 (1 ng/mL) on the *I*_f_ current confirmed an increase in channel maximal conductance (Figure 6D), and, differently from mouse cells, a positive shift of the activation curve was elicited. A clear explanation for this difference is still elusive; however, it likely arises from differences in the species (mouse vs. human) and/or from the immature state of the pCMs model.

Data reported in Figure 7 support the hypothesis that the FGF23‐induced increase in *I*_f_ *g*_max_ is at least partly mediated by transcriptional upregulation of HCN2 and HCN4 that occurs after 48 h. Although additional experiments are required to fully define the signaling pathway activated by FGF23, our current observations suggest a possible involvement of ERK signaling. In particular, the partial colocalization between FGFR1 and Klotho (Figure 5C,D), together with the FGF23‐dependent increase in ERK phosphorylation (Figure S5), is consistent with the idea that in pacemaker cells the effects of FGF23 may be mediated through FGFR1–Klotho‐dependent ERK activation.

The Ca^2+^‐clock mechanism is also considered a relevant contributor to SAN automaticity [24], however, the lack of effects of FGF23 on the *I*_CaL_ current and on the AP THR reported in our experiment (Figures 3E–H, 2C, and Tables 1 and 2) would exclude the Ca^2+^‐clock mechanism as a possible FGF23 target [21].

Homozygous defective α‐Klotho (*kl/kl*) mice display intrinsic bradyarrhythmia and cardiac fragility since, when exposed to restraining stress, they die of sinus arrest [13]. *kl/kl* SAN cells display a decreased *I*_f_ current density compared to their WT littermates (Figure 4A), and this may explain, at least in part, the intrinsic bradycardia characterizing these mice. Although our data (Figure 4B,C) suggest that the dysfunctional SAN phenotype of *kl/kl* mice may arise from a defective FGF23/Klotho pathway, the mechanism may be more complex and possible additional mechanisms can include a decrease availability of the soluble Klotho form and/or aspecific effects of FGF23, whose levels are high in these mice [12]. Indeed, it is known that soluble α‐Klotho (sKlotho) can regulate the membrane density of several ion channels and transporters by modifying protein glycosylation, through its glycosidase and sialidase activities, and by regulating membrane lipid raft structure and function [25, 26]. These mechanisms may thus contribute to the modulation of the *I*_f_ current since all four HCN isoforms (the molecular correlate of the pacemaker current) contain a conserved extracellular consensus site for *N*‐glycosylation, and, as many other membrane proteins, they are often localized in and influenced by lipid rafts [27, 28, 29]. *kl/kl* mice have extremely high levels of circulating FGF23 (about 460 ng/mL) [12] which may elicit additional still unidentified effects likely resulting from FGFR family members not associated with the α‐Klotho co‐factor. Finally, the lack of the cardiac protective function of soluble klotho (i.e., prevention of fibrosis, anti‐inflammatory, and antioxidant [4]) should also be considered. Further experiments are required to shed light on the mechanisms associated with SAN dysfunctions in *kl/kl* mice.

The evidence that the FGF23/FGFR1/α‐Klotho signaling is present in both mouse SAN cells and in human pCM suggests that the SAN is a target of the endocrine action of FGF23. In particular, the presence of the co‐factor α‐Klotho increases the binding affinity and thus ensures activation at physiological concentrations [1, 13]. Thus, the FGF23‐SAN link may represent an important player in the cardio‐renal axis by coupling calcium and phosphate homeostasis to HR; dysregulation of this modulatory mechanism may result in SAN dysfunction. Our in vitro data suggest that elevated plasma levels of FGF23 may lead to an increased HR. However, patients with pathological conditions associated with increasing levels of FGF23, such as end‐stage renal disease, chronic heart failure, and hereditary hypophosphatemia, can present either sinus tachycardia or bradycardia [1, 13, 30, 31, 32, 33, 34]. It is reasonable to speculate that this complexity may arise from the simultaneous influence of additional factors that can modulate the chronotropic activity of the SAN, such as aging, autonomic dysfunction, diabetes, hypertension, cardiac hypertrophy, systemic inflammation, and parathyroid hormone plasma levels [35, 36, 37, 38].

## 4 Conclusion

In conclusion, the relevance of the SAN in setting HR makes it an important target of several neurohumoral agents, including neurotransmitters, neuropeptides, autocrine, paracrine, and endocrine factors that act in concert to allow a rapid adaptation of the HR to different body needs; dysregulation of these factors can promote SAN dysfunction [37]. Our data indicate that FGF23 can be added to the list of these agents, and potential pathological consequences may arise in contexts of chronically or markedly elevated FGF23 levels.

## 5 Limitations of the Study

Experimental models used in this study present intrinsic limitations that must be acknowledged. Maintaining SAN tissue in an artificial culture environment may favor cell “remodeling” thus potentially introducing experimental bias.

Similarly, *kl/kl* mice are known to exhibit a severe systemic illness, a condition that may also lead to secondary alterations potentially influencing the results. As originally described, a severe reduction of Klotho expression in mice results in a syndrome that closely resembles human aging, characterized by reduced lifespan, infertility, arteriosclerosis, skin atrophy, osteoporosis, and emphysema [3]. These animals had been extensively characterized in previous studies, which consistently documented alterations in both macroscopic and biochemical parameters [12, 39]. In particular, *kl/kl* mice at 6–8 weeks of age display reduced body weight (BW), heart weight (HW), and kidney weight, together with an increased HW/BW ratio and elevated levels of urea, blood urea nitrogen, and circulating FGF23 compared with their wild‐type littermates. Moreover, no relevant changes in plasma phosphate levels were observed between the two groups, probably due to the compensatory action of FGF23 as a phosphaturic hormone, whose levels are typically markedly elevated in kl/kl mice. Nevertheless, other factors related to the local environment and feeding regimens in animal facilities may influence these measurements.

The use of hiPSC‐derived cardiomyocytes, obtained with our protocol, also carries constraints. These cells retain an immature electrophysiological phenotype, and although we did not perform a formal quantification in this system, the presence of immature ventricular, atrial, and fibroblast‐like cells is expected. A hallmark of this immaturity is the persistence of spontaneous pacemaker activity, driven by sustained high expression of the *I*_f_ current and very low levels of IK_1_. Notably, these same features are defining electrophysiological traits of sinoatrial node cells. As suggested by Giannetti et al. [40], hiPSC‐derived cardiomyocytes may therefore still provide a valuable platform for studying human pacemaker activity; consequently, we referred to these cells as human pacemaker‐like cardiac cells.

Despite the specific limitations associated with each model, SAN tissue incubation and pCMs consistently yielded comparable results. This convergence strengthens the robustness of our findings and supports the use of these complementary in vitro approaches to dissect the direct effects of FGF23 while minimizing the confounding influences inherent to in vivo systems.

## 6 Materials and Methods

### 6.1 Animals

All experiments have been performed on SAN cells/tissues isolated from 2 to 3 month‐old male C57Bl/6J mice, and 2 month‐old male and female hypomorphic α‐Klotho (*kl/kl*) mice and their wild‐type littermates (*+/+*). C57Bl/6J procedures conformed to European and Italian laws (2010/63/UE D. and Lgs n. 2014/26), and were approved by the Animal Welfare Body of the Università degli Studi di Milano and by the Italian Minister of Health. *Kl/kl* is a transgenic strain generated by Prof. Makoto Kuro‐o [3]. *kl/kl* mice showed a severe reduction in the transcription of *Kl* gene and for that they were defined hypomorfic. This defect in Klotho expression results in a syndrome that resemble human aging as described in [3]. *Kl/kl* strain procedures conformed to European and Spanish laws, and were approved by the Bioethical Committee of the Universidad Autónoma de Madrid, and by the General Direction of Agriculture and the Environment at the Environment Council of Madrid (PROEX 186.5/20).

### 6.2 Statistical Analysis

Normality of data distributions was verified by the Shapiro–Wilk test. Normally distributed data were analyzed for statistical significance using Student's pair *t*‐test (correlated samples), Student's *t*‐test (uncorrelated samples), or one‐way ANOVA followed by Bonferroni post hoc test (multiple comparisons). Non‐normally distributed data were analyzed for statistical significance using the nonparametric Mann–Whitney *U*‐test or the Kruskal–Wallis test (followed by Dunn's test). A *p* < 0.05 was considered statistically significant.

Additional methods are provided in the Supporting Information.

## Author Contributions

**Makoto Kuro‐o:** writing – review and editing. **Giorgia Bertoli:** investigation, formal analysis, writing – original draft, writing – review and editing. **Gema Ruiz‐Hurtado:** conceptualization, writing – review and editing, funding acquisition. **David Molla:** investigation. **Carmen Delgado:** conceptualization, writing – review and editing. **Mirko Baruscotti:** conceptualization, writing – review and editing, methodology. **Raffaella Milanesi:** investigation. **Serena Canzolino:** investigation. **Patrizia Benzoni:** investigation, writing – review and editing, formal analysis. **Annalisa Bucchi:** conceptualization, methodology, formal analysis, writing – original draft, supervision, writing – review and editing, funding acquisition, project administration, validation, data curation. **Chiara Piantoni:** investigation.

## Funding

This work is supported by, LINEA 2 Dipartimento di Bioscienze, Università degli Studi di Milano to AB, Instituto de Salud Carlos III (ISCIII) (IFEQ21/00012, PI23/00182) co‐funded by the European Union, Ministry of Science and Innovation and by the European Union “NextGenerationEU”/PRTR (CNS2023‐144891), Biomedicine Network Comunidad de Madrid (P2022/BMD‐7223 CIFRA_COR‐CM), and the Spanish Society of Cardiology (SEC)/Spanish Foundation of Heart (SEC/FEC‐INV‐BAS 23) to G. R‐H.

## Conflicts of Interest

The authors declare no conflicts of interest.

## Supporting information


**Table S1:** Primers list. Forward (F) and Reverse (R).
**Table S2:** Primers list. Primer used for the RT‐PCR; Forward (F) and Reverse (R).
**Figure S1:** Representative co‐staining of sinoatrial node (SAN) cells (A) and pacemaker cardiomyocytes (pCMs) (B) with anti‐HCN4 (yellow) and anti‐αKlotho (ab1, SAB3500604 magenta) antibodies with (bottom) and without (top) the specific blocking peptide for the anti‐αKlotho antibody. (C) Top: Representative co‐staining of pCM with FGFR1 (yellow) and anti‐αKlotho (ab2, AF1819, magenta). Bottom: Negative controls. The goat IgG isotype antibody was used as negative control for ab2. Nuclei were stained Hoechst (blue). Scale bar 10μm.
**Figure S2:** FGF23 does not affect action potentials (AP) rate and I_f_ of SAN cells after short‐term exposure (A). Mean (black circle) ± SEM and single AP rate values obtained before (open circles) and at the end (open squares) of FGF23 10 ng/mL SAN cells perfusion for 74–120 s. Control: 8.23 ± 0.54 Hz; FGF23: 8.08 ± 0.68 Hz;*n* = 5/3 mice, p > 0.05 pair‐sample t‐test. (B) Left: Sample I_f_ traces recorded at −75/−125 mV (hp = −30 mV) in the absence and in the presence of FGF23 10 ng/mL as indicated. Right: Mean ± SEM of normalized current amplitudes at −75 and −125 mV (*n* = 4/2 mice, p > 0.05, One sample t‐test) (C) Mean (black circle) ± SEM and single AP rate values obtained after 30–60 min incubation of SAN cells with either vehicle (open circles) or FGF23 10 ng/mL (open squares). Control: 8.45 ± 0.75 Hz,*n* = 9/6 mice; FGF23: 8.71 ± 0.61 Hz;*n* = 8/6 mice (p > 0.05 sample t‐test). (D) Mean (filled circle) ± SEM and single gf values. (E) mean I_f_ activation curves obtained after 30–60 min incubation of SAN cells with either vehicle (open circles) or FGF23 10 ng/mL (open squares).
**Figure S3:** Heterogeneity of mouse SAN action potentials. Overlays of individual AP waveforms (*n* = 8) obtained after 48 h SAN incubation with vehicle.
**Figure S4:** (A) RT‐PCR analysis of α‐Klotho (Kl) and FGFR1‐4 (R1‐4) mRNAs in two different lines of pCMs. Negative control (no cDNA) is also shown. RT‐PCR analysis amplified transcripts of all members of the FGFR family and the co‐receptor α‐Klotho. For α‐Klotho, in addition to the expected band (163 bp), we identified one/two additional longer transcripts that may represent alternative nonfunctional klotho transcripts.6.
**Figure S5:** Western blot analysis for total ERK and phospho ERK (P‐ERK) expression in pCMs treated for 48 h with vehicle (control, 3 samples S4, S5, and S6) or FGF23 1 ng/ml (3 samples S1, S2, and S3). Caveolin 1 (CAV1) has been used as reference. (B) Plot of intensity blot analysis for ERK tot, P‐ERK and P‐ERK/ERKtot ratio, normalized to CAV1. FGF23 induced a significant increase in the P‐ERK/ERK tot ratio (*p < 0.05 Student t‐test).

## Data Availability

The data that support the findings of this study are available from the corresponding author upon reasonable request.

## References

[1] R. G. Erben , “Physiological Actions of Fibroblast Growth Factor‐23,” Frontiers in Endocrinology 9, no. MAY (2018): 267. 29892265 10.3389/fendo.2018.00267 PMC5985418

[2] R. Marsell and K. B. Jonsson , “The Phosphate Regulating Hormone Fibroblast Growth Factor‐23,” Acta Physiologica (Oxf) 200, no. 2 (2010): 97–106. 10.1111/j.1748-1716.2010.02163.x 20618171

[3] M. Kuro‐o , Y. Matsumura , H. Aizawa , et al., “Mutation of the Mouse Klotho Gene Leads to a Syndrome Resembling Ageing,” Nature 390, no. 6655 (1997): 45–51. 9363890 10.1038/36285

[4] G. J. Prud'homme , M. Kurt , and Q. Wang , “Pathobiology of the Klotho Antiaging Protein and Therapeutic Considerations,” Frontiers in Aging 3 (2022): 931331. 35903083 10.3389/fragi.2022.931331 PMC9314780

[5] H. Kurosu , Y. Ogawa , M. Miyoshi , et al., “Regulation of Fibroblast Growth Factor‐23 Signaling by Klotho,” Journal of Biological Chemistry 281, no. 10 (2006): 6120–6123. 16436388 10.1074/jbc.C500457200 PMC2637204

[6] I. Urakawa , Y. Yamazaki , T. Shimada , et al., “Klotho Converts Canonical FGF Receptor Into a Specific Receptor for FGF23,” Nature 444, no. 7120 (2006): 770–774. 17086194 10.1038/nature05315

[7] T. V. Grigore , M. Zuidscherwoude , H. Olauson , and J. G. Hoenderop , “Lessons From Klotho Mouse Models to Understand Mineral Homeostasis,” Acta Physiologica 240, no. 10 (2024): e14220. 39176993 10.1111/apha.14220

[8] J. A. Navarro‐García , M. Fernández‐Velasco , C. Delgado , et al., “PTH, Vitamin D, and the FGF‐23–Klotho Axis and Heart: Going Beyond the Confines of Nephrology,” European Journal of Clinical Investigation 48, no. 4 (2018): e12902. 10.1111/eci.12902 29394451

[9] S. Vázquez‐Sánchez , J. Poveda , J. A. Navarro‐García , et al., “An Overview of FGF‐23 as a Novel Candidate Biomarker of Cardiovascular Risk,” Frontiers in Physiology 12 (2021): 632260. 33767635 10.3389/fphys.2021.632260 PMC7985069

[10] M. Leifheit‐Nestler and D. Haffner , “Paracrine Effects of FGF23 on the Heart,” Frontiers in Endocrinology 9, no. MAY (2018): 1–13. 29892269 10.3389/fendo.2018.00278 PMC5985311

[11] A. Grabner , A. P. Amaral , K. Schramm , et al., “Activation of Cardiac Fibroblast Growth Factor Receptor 4 Causes Left Ventricular Hypertrophy,” Cell Metabolism 22, no. 6 (2015): 1020–1032. 26437603 10.1016/j.cmet.2015.09.002 PMC4670583

[12] J. A. Navarro‐García , A. Rueda , T. Romero‐García , et al., “Enhanced Klotho Availability Protects Against Cardiac Dysfunction Induced by Uraemic Cardiomyopathy by Regulating Ca^2+^ Handlingtle,” British Journal of Pharmacology 177, no. 20 (2020): 4701–4719. 32830863 10.1111/bph.15235 PMC7520447

[13] K. Takeshita , T. Fujimori , Y. Kurotaki , et al., “Sinoatrial Node Dysfunction and Early Unexpected Death of Mice With a Defect of Klotho Gene Expression,” Circulation 109, no. 14 (2004): 1776–1782. 15037532 10.1161/01.CIR.0000124224.48962.32

[14] S. K. Teixeira , R. Pontes , L. F. G. Zuleta , et al., “Genetic Determinants of Blood Pressure and Heart Rate Identified Through ENU‐Induced Mutagenesis With Automated Meiotic Mapping,” Science Advances 10, no. 9 (2024): eadj9797. 38427739 10.1126/sciadv.adj9797 PMC10906923

[15] M. R. Boyett , J. Yanni , J. Tellez , et al., “Regulation of Sinus Node Pacemaking and Atrioventricular Node Conduction by HCN Channels in Health and Disease,” Progress in Biophysics and Molecular Biology 166 (2021): 61–85. 34197836 10.1016/j.pbiomolbio.2021.06.008

[16] C. Faul , A. P. Amaral , B. Oskouei , et al., “FGF23 Induces Left Ventricular Hypertrophy,” Journal of Clinical Investigation 121, no. 11 (2011): 4393–4408. 21985788 10.1172/JCI46122 PMC3204831

[17] C. D. Touchberry , T. M. Green , V. Tchikrizov , et al., “FGF23 Is a Novel Regulator of Intracellular Calcium and Cardiac Contractility in Addition to Cardiac Hypertrophy,” American Journal of Physiology. Endocrinology and Metabolism 304, no. 8 (2013): E863–E873. 23443925 10.1152/ajpendo.00596.2012 PMC3625783

[18] S. Y. Huang , Y. C. Chen , Y. H. Kao , et al., “Fibroblast Growth Factor 23 Dysregulates Late Sodium Current and Calcium Homeostasis With Enhanced Arrhythmogenesis in Pulmonary Vein Cardiomyocytes,” Oncotarget 7, no. 43 (2016): 69231–69242. 27713141 10.18632/oncotarget.12470 PMC5342473

[19] C. H. Peters , C. Rickert , S. Morotti , et al., “The Funny Current if Is Essential for the Fight‐Or‐Flight Response in Cardiac Pacemaker Cells,” Journal of General Physiology 154, no. 12 (2022): e202213193. 36305844 10.1085/jgp.202213193 PMC9812006

[20] C. Rickert and C. Proenza , “ParamAP: Standardized Parameterization of Sinoatrial Node Myocyte Action Potentials,” Biophysical Journal 113, no. 4 (2017): 765–769. 28834713 10.1016/j.bpj.2017.07.001 PMC5567588

[21] A. Bucchi , M. Baruscotti , R. B. Robinson , and D. DiFrancesco , “Modulation of Rate by Autonomic Agonists in SAN Cells Involves Changes in Diastolic Depolarization and the Pacemaker Current,” Journal of Molecular and Cellular Cardiology 43, no. 1 (2007): 39–48. 17543331 10.1016/j.yjmcc.2007.04.017

[22] M. E. Mangoni , B. Couette , E. Bourinet , et al., “Functional Role of L‐Type Cav1.3 Ca2+ Channels in Cardiac Pacemaker Activity,” Proceedings of the National Academy of Sciences of the United States of America 100, no. 9 (2003): 5543–5548. 12700358 10.1073/pnas.0935295100 PMC154381

[23] S. Landi , F. Giannetti , P. Benzoni , et al., “Lack of the Transcription Factor Nfix Causes Tachycardia in Mice Sinus Node and Rats Neonatal Cardiomyocytes,” Acta Physiologica (Oxford, England) 239, no. 2 (2023): e13981. 37186371 10.1111/apha.13981

[24] E. Carmeliet , “Pacemaking in Cardiac Tissue. From IK2 to a Coupled‐Clock System the IK2 Hypothesis,” Physiological Reports 7, no. 1 (2019): e13862. 30604930 10.14814/phy2.13862 PMC6317064

[25] C. L. Huang , “Regulation of Ion Channels by Secreted Klotho: Mechanisms and Implications,” Kidney International 77, no. 10 (2010): 855–860. 20375979 10.1038/ki.2010.73

[26] G. Dalton , S.‐W. An , S. I. Al‐Juboori , et al., “Soluble Klotho Binds Monosialoganglioside to Regulate Membrane Microdomains and Growth Factor Signaling,” Proceedings of the National Academy of Sciences of the United States of America 114, no. 4 (2017): 752–757. 28069944 10.1073/pnas.1620301114 PMC5278494

[27] B. Much , C. Wahl‐Schott , X. Zong , et al., “Role of Subunit Heteromerization and N‐Linked Glycosylation in the Formation of Functional Hyperpolarization‐Activated Cyclic Nucleotide‐Gated Channels*,” Journal of Biological Chemistry 278, no. 44 (2003): 43781–43786. 12928435 10.1074/jbc.M306958200

[28] A. Barbuti , B. Gravante , M. Riolfo , R. Milanesi , B. Terragni , and D. DiFrancesco , “Localization of Pacemaker Channels in Lipid Rafts Channel Kinetics,” Circulation Research 94, no. 10 (2004): 1325–1331. 15073040 10.1161/01.RES.0000127621.54132.AE

[29] G. Campostrini , M. Bonzanni , A. Lissoni , et al., “The Expression of the Rare Caveolin‐3 Variant T78M Alters Cardiac Ion Channels Function and Membrane Excitability,” Cardiovascular Research 113, no. 10 (2017): 1256–1265. 28898996 10.1093/cvr/cvx122 PMC5852518

[30] L. N. Zhang , G. Yang , C. Cheng , et al., “Plasma FGF23 Levels and Heart Rate Variability in Patients With Stage 5 CKD,” Osteoporosis International 26, no. 1 (2015): 395–405. 25224292 10.1007/s00198-014-2862-7

[31] R. S. Elzayat , W. A. Bahbah , R. S. Elzaiat , and B. A. Elgazzar , “Original Research: Fibroblast Growth Factor 23 in Children With or Without Heart Failure: A Prospective Study,” BMJ Paediatrics Open 7, no. 1 (2023): e001753. 36828640 10.1136/bmjpo-2022-001753 PMC9972412

[32] R. B.‐N. S. R. L. R. Espersen , “Heart Rate and Blood Pressure, but Not Pulse Wave Velocity, Are Higher Among Adults With Hereditary Hypophosphatemia Compared to Healthy Controls: A Cross‐Sectional Study,” Endocrine Abstracts 90 (2023): P33.

[33] M. C. G. Wong , J. M. Kalman , E. Pedagogos , et al., “Temporal Distribution of Arrhythmic Events in Chronic Kidney Disease: Highest Incidence in the Long Interdialytic Period,” Heart Rhythm 12, no. 10 (2015): 2047–2055. 26111801 10.1016/j.hrthm.2015.06.033

[34] J. Rautavaara , T. Kerola , K. Kaartinen , et al., “Asystole Episodes and Bradycardia in Patients With End‐Stage Renal Disease,” Nephrology, Dialysis, Transplantation 37, no. 3 (2022): 575–583. 10.1093/ndt/gfab023 33527131

[35] C. Faul , “FGF23 Effects on the Heart‐Levels, Time, Source, and Context Matter,” Kidney International 94, no. 1 (2018): 7–11. 29933856 10.1016/j.kint.2018.03.024

[36] N. Sathnur , E. Ebin , and D. G. Benditt , “Sinus Node Dysfunction,” Cardiology Clinics 41, no. 3 (2023): 349–367. 37321686 10.1016/j.ccl.2023.03.013

[37] E. A. MacDonald , R. A. Rose , and T. A. Quinn , “Neurohumoral Control of Sinoatrial Node Activity and Heart Rate: Insight From Experimental Models and Findings From Humans,” Frontiers in Physiology 11 (2020): 170. 32194439 10.3389/fphys.2020.00170 PMC7063087

[38] S. P. Whelton , V. Narla , M. J. Blaha , et al., “Association Between Resting Heart Rate and Inflammatory Biomarkers (High‐Sensitivity C‐Reactive Protein, Interleukin‐6, and Fibrinogen) (From the Multi‐Ethnic Study of Atherosclerosis),” American Journal of Cardiology 113, no. 4 (2014): 644–649. 24393259 10.1016/j.amjcard.2013.11.009 PMC4280910

[39] J. A. Navarro‐García , R. Salguero‐Bodes , L. González‐Lafuente , et al., “The Anti‐Aging Factor Klotho Protects Against Acquired Long QT Syndrome Induced by Uremia and Promoted by Fibroblast Growth Factor 23,” BMC Medicine 20, no. 1 (2022): 1–19. 35042527 10.1186/s12916-021-02209-9 PMC8767669

[40] F. Giannetti , P. Benzoni , G. Campostrini , et al., “A Detailed Characterization of the Hyperpolarization‐Activated Funny Current (If) in Human‐Induced Pluripotent Stem Cell (iPSC)‐Derived Cardiomyocytes With Pacemaker Activity,” European Journal of Physiology 473, no. 7 (2021): 1009–1021. 33934225 10.1007/s00424-021-02571-w PMC8245366

