# Supplementary material for: The Binding of Fibroblast Growth Factor 23 (FGF23) to FGF Receptor‐αKlotho Complex Increases Sinoatrial Electrical Activity
Source: Acta Physiol (Oxf). 2026 Sep 1;242(10):e70293. doi: 10.1111/apha.70293 (PMC13531400; doi:10.1111/apha.70293)
Supplement: Supplementary file 1 [file APHA-242-e70293-s001.docx]

**Supplementary materials**

**The binding of Fibroblast Growth Factor 23 (FGF23) to FGF receptor-αKlotho complex increases sinoatrial electrical activity**

Giorgia Bertoli, Patrizia Benzoni, Serena Canzolino, David Molla, Raffaella Milanesi, Chiara Piantoni, Makoto Kuro-o, Carmen Delgado, Gema Ruiz-Hurtado, Mirko Baruscotti, Annalisa Bucchi

**Materials and Methods**

**Mouse sinoatrial cells isolation**

Mice were sacrificed by cervical dislocation and hearts were quickly extracted and immersed in Tyrode solution (mM: NaCl, 140; KCl, 5.4; CaCl_2_, 1.8; MgCl_2_, 1; D-glucose, 5.5; Hepes-NaOH, 5; pH 7.4) containing heparin (10 U/mL, Sigma-Aldrich). The SAN tissue was dissected, and SAN myocytes were isolated using a previously described enzymatic and mechanical protocol^1^. The enzymatic solution contained collagenase IV (224 U/mL; Worthington), elastase (1.4 U/mL; Sigma-Aldrich), and protease (0.41 U/mL; Sigma-Aldrich).

For long-term treatment, SAN cells isolation was performed after incubation of the whole SAN for 48hrs with vehicle or recombinant mouse FGF23 (FGF23, R&D systems) or PD173074 (Selleckchem) or FGF23+ PD173074. The SAN tissues after dissection were placed in a petri dish coated with SYLGARD 184 elastomer kit and fixed with four pins at the level of the superior and inferior vena cava, crista terminalis, and interatrial septum. SAN tissues were maintained in medium 199 (Sigma Aldrich) complemented with Fetal Bovine Serum (FBS) 20% (GIBCO), Insulin-Transferrin-Sodium selenite (ITS) 1% (Sigma Aldrich), 2,3 ButaneDione Monoxime (BDM) 10 mM (Sigma Aldrich) and antibiotic at 37°C, 5% of CO_2_. Vehicle (PBS with 0.1% Bovine Serum Albumin), FGF23, and PD1730274 were added to the medium. The medium was changed after 24 hrs.

Cells were isolated immediately after SAN dissection to perform the 30min/1 hr incubation. For acute experiments, freshly isolated or 48hrs vehicle-treated cells were superfused with FGF23 10 ng/mL.

The cells were kept at 4 °C in Tyrode solution for the day of experiments, while, during 30-60 min incubation the cells were plated in 35 mm Petri dish with FGF23 10 ng/mL or vehicle (Tyrode 0.1% BSA) at room temperature in Tyrode solution.

**Human induced pluripotent stem cells-derived pacemaker-like cardiomyocytes (pCMs)**

We used previously characterized and published human induced pluripotent stem cells lines (hiPSC) obtained from healthy donors^2^. hiPSCs were cultured onto Matrigel-coated plates in TeSR-E8 medium (Stem Cell Technologies). Cardiac differentiation was induced starting from a sub-confluent layer of iPSC cultures using the Cardiomyocyte Differentiation Kit (Thermo Fisher Scientific) according to the protocol reported in^2^. On day 13 cells were incubated with either vehicle or FGF23, and after 48 hrs (day 15th of differentiation) pCMs were isolated with TrypLE express enzyme (Thermo Fisher Scientific) and plated on fibronectin (Corning) coated dish for patch-clamp experiments. The recombinant human FGF23 (Sigma-Aldrich) was used for all the experiments involving pCMs. pCMs obtained from independent differentiation rounds of the same hiPSC line (pCMs_2 in suppl Fig 4) were used for the patch‑clamp, RT‑qPCR, immunolabelling, and western blot experiments. For RT-PCR an additional hiPSC line was analysed after differentiation into pCMs (pCMs_1 in suppl Fig 4)

**Reverse transcriptase (RT) quantitative PCR (qPCR) analysis**

For both mouse SAN and pCMs samples total mRNA was isolated using TRIzol reagent (Thermo Fisher Scientific). After the extraction, 1 µg of mRNA was reverse transcribed using a commercial KIT (Maxima First Strand cDNA Synthesis; Thermo Fisher Scientific). qPCR was performed in triplicate using 20 ng for mouse sample, and 10 ng for pCMs of cDNA with Maxima SYBR Green qPCR Master Mix (Thermo Fisher Scientific) and specific primers in a Real-Time PCR detection system (gene 9600 Plus, BIOER. Expression data were analyzed using 2^(-ΔCT) method relative to expression level of housekeeping gene α-actin for mouse sample, and troponin (TNNT) or RLP0 for pCMs. Primers used for RT-PCR are listed **in Table S1**. Total mRNA was extracted from SAN tissues frozen immediately after isolation from C57Bl/6J mice hearts; 2 or 3 SAN tissues were used for each sample.

**Table S1. Primers list.** Forward (F) and Reverse (R).

| Gene | RTqPCR (mouse) | RTqPCR (human) |
| --- | --- | --- |
| α-Klotho | **F_**ACGTTCAAGTGGACACTACTCT  **R_**GGTTATCTGAGGCCGGATGG | **F**_AATGGAATCGATGACGGGCT  **R**_ATGTGGGCTTTGAGAGCTTCG |
| FGFR1 | **F_**ACTCTGCATGGTTGACCGTT  **R_**AGGTAGAGCGGTGAGGTCAT | **F**_ACTCTGCATGGTTGACCGTT  **R**_TTGTAGACGATGACCGACCC |
| FGFR2 | **F**_TGTGGTCCCGTCAGACAAAG  **R**_TGGGCATCGCTGTAAACCTT | **F**_TGGAAAGTGTGGTCCCATCTG  **R**_ACTCTACGTCTCCTCCGACC |
| FGFR3 | **F_**CTACTTCCGAGCCTCCTGGT  **R_**CATCCTTAGCCCAGACCGTG | **F**_CTCGGGAGATGACGAAGACG  **R**_CGGGCCGTGTCCAGTAAG |
| FGFR4 | **F**_ATCAGGCTCACTGGTTCTGC  **R_**TCCAAAGAGAACTGTCGGGC | **F**_TGCTCAAAGACAACGCCTCT  **R_**GCACTCCACGATCACGTACA |
| α-actin | **F_**AAACTGTGTTACGTCGCCCTG  **R_**GCGGATATCGATGTCACACTTC |  |
| HCN1 |  | **F_**TGAAGCTGACAGATGGCTCTT  **R_**CTGGCAGTACGACGTCCTTT |
| HCN2 |  | **F_**CTGATCCGCTACATCCATCA  **R_**AGATTGCAGATCCTCATCACC |
| HCN4 |  | F_ AACAGGAGAGGGTCAAGTCG  **R_**ATCAGGTTTCCCACCATCAG |
| TNNT2 |  | **F_**AAGCCCAGGTCGTTCATGCCC  **R_**CTCCATGCGCTTCCGGTGGA |
| RLP0 |  | **F_** ATCCAGCAGGTGTTCGACAAT  **R_**GCCAGGACTCGTTTGTACCC |

**Reverse transcriptase PCR (RT-PCR) analysis**

The mRNA from pCMs was extracted with Direct-zol RNA mini prep KIT (ZYMO RESEARCH) and reverse transcribed using Maxima First Strand cDNA Synthesis KIT (Thermo Fisher Scientific). RT-PCR was performed using 40 ng of cDNA with PCR master mix FastStart Taq Polymerase (ROCHE). Primers used for RT-PCR are listed in **Table S2**. The expected lengths of the PCR products were (bp): 163 α-Klotho, 139 FGFR1, 174 FGFR2, 175 FGFR3, and 153 FGFR4; the correct sequence of the expected bands was confirmed by sequencing.

**Table S2. Primers list.** Primer used for the RT-PCR; Forward (F) and Reverse (R).

| Gene | RT-PCR (human) |
| --- | --- |
| α-Klotho | **F**_TCAGCCCCTAGAAGGGACAT  **R**_CTTGGTCACAACCCCATCCA |
| FGFR1 | **F**_TTGTAGACGATGACCGACCC  **R**_TTGTAGACGATGACCGACCC |
| FGFR2 | **F**_TGGAAAGTGTGGTCCCATCTG  **R**_ACTCTACGTCTCCTCCGACC |
| FGFR3 | **F**_CTCCTCGGAGTCCTTGGGGA  **R**_GCCATCCTTGACCCAGACAG |
| FGFR4 | **F**_ATCCTGTACGCGTCGGGCTC  **R_**CTCCAGGGAGAACTGTCGGG |

## **Immunofluorescence analysis**

Immunofluorescence was performed in non-treated pCMs and murine SAN cells. The cells were fixed with 4 % paraformaldehyde in phosphate-buffered saline (PBS) and treated with PBS containing Triton X-100 0.3% (Sigma Aldrich); MgCl_2,_ 3 mM; and donkey serum 10% (Abcam). Primary antibodies used were: Rabbit anti- α-Klotho (SAB3500604, Sigma-Aldrich) 1∶200; Rabbit anti-FGF Receptor 1 ([ZRB1243](https://www.sigmaaldrich.com/IT/it/product/sigma/zrb1243), Merck) 1∶50; Rabbit anti-FGF Receptor 2 (Ab10648, Abcam) 1∶100; Rat anti-HCN4 (MA3-903, Thermo Fisher Scientific) 1∶100. The specificity of anti-klotho antibody was assessed by using the specific blocking peptide ([SBP3500604](https://www.sigmaaldrich.com/IT/it/product/sigma/sbp3500604), Merck Sigma-Aldrich; **Fig 1S**). The anti‑Klotho antibody alone, as well as the antibody with the blocking peptide (in a ratio 1:10), were incubated in PBS containing 5% donkey serum at 4 °C for approximately 24 h before use. Co‑immunostaining for α-Klotho and FGFR1 was performed using the goat anti‑ α-Klotho antibody (1:20; AF1819 R&D system), with goat IgG (1:20; AB-108-C, R&D Systems) as the isotype control.

Secondary antibodies used were: Alexa fluor 594 donkey anti-rabbit antibody (Invitrogen), Alexa flour 488 donkey anti-rat antibody (Invitrogen), Alexa fluor 594 donkey anti-goat antibody (Invitrogen), and Alexa flour 488 donkey anti-rabbit antibody (Invitrogen) . Nuclei were stained with either DAPI (0,5 µg/ml) or Hoechst (1 µg/ml). Images were acquired with a Video Confocal microscopy (ViCo Nikon) or Nikon Spinning Disk.

To ensure optimal visualization for publication, brightness and contrast settings were adjusted in Image J using the secondary-only and α-Klotho negative controls as a reference.

**Patch-clamp experiments**

Patch-clamp experiments were performed in the whole-cell configuration at 35±0.5 °C for mouse cells and 36±0.5 °C for pCMs. For Action Potential (AP) and If current recordings in mouse cells patch pipettes were filled with (mM): K-Aspartate, 130; NaCl, 10; EGTA-KOH, 5; CaCl_2_, 2; MgCl_2_, 2; ATP (Na-salt), 2; creatine phosphate, 5; GTP (Na-salt), 0.1; pH=7.2. For I_CaL_ measurements patch pipettes were filled with (mM): CsCl, 135; MgCl_2_, 1; ATP- (Na-salt), 4; GTP (Na-salt), 0.1; EGTA-KOH, 5; Hepes-KOH, 5; pH 7.2. For AP and If current recordings in pCMs patch pipettes were filled with (mM): KCl, 120; Hepes-NaOH, 20; EGTA-KOH, 0.1; MgCl_2_, 2; ATP (Mg-salt), 10; pH 7.1. External solutions were: Tyrode for mouse AP recordings; Tyrode solution with BaCl_2_ (1 mM) and MnCl_2_ (2 mM) for mouse If recordings; Tyrode high glucose solution (in mM: NaCl, 137; KCl, 5; CaCl_2_, 2; MgCl_2_, 1; D-glucose, 10; Hepes-NaOH, 10; pH 7.4) for pCMs AP recordings; Tyrode high glucose solution with BaCl_2_ (1 mM) and MnCl_2_ (2 mM) for pCMs If recordings. I_CaL_ was measured as Nifedipine-sensitive current superfusing cells with a solution without/with Nifedipine 10 μM containing (mM): NMDG, 130; CaCl_2_, 2; MgCl_2_, 1; 4-amino-pyridine, 10; Hepes, 25; tetrodotoxin (TTX; Hello-bio,10 μM), pH=7,4.

APs were recorded in current-clamp configuration from single cells or small uniformly-beating cell aggregates and analyzed using the AP program^3^, the program described in^4^ and Clampfit. The current-voltage relationship (I/V) and activation curve of the If current were obtained by applying a step protocol with a first step to a test voltage in the range -30/-135 mV (-15 mV decrement), a second step to -125 mV and a third step to 10 mV, holding potential (hp) =-30mV. Each step was long enough to reach steady-state current activation. Normalized tail currents at -125 mV were fitted to the Boltzmann distribution: y = (1/(1 + exp(V − V_1/2_)/s)), where V is voltage, y fractional activation, V_1/2_ the half-activation voltage, and s the inverse-slope factor. The maximal conductance (g_fmax_) was obtained from the IV curves as the slope of the linear fit of the fully activated current densities (-120 and -135 mV). The steady state IV curves were obtained by fitting the data points as described in^5^. The acute effect of FGF23 on If current was evaluated by applying a double-pulse protocol which allows to observe the effect on the current both near the half-activation voltage (-75 mV) and at full activation (-125 mV). Experimental dose-response points were fitted to the Hill equation (y = y_max_/(1+(k/x)h)), where ymax is the maximal effect, x is the drug concentration, k is the drug concentration eliciting half-maximal effect.

I_CaL_ current was recorded in single SAN myocytes; the I/V relationship and activation curve of I_CaL_ were determined by applying 150 msec depolarizing steps ranging from -60 to +50 mV (10 mV increment) from a holding potential (hp) of -60 mV in the absence and in the presence of nifedipine. For inactivation, a step at 0 mV (300 msec) was applied after preconditioning voltage steps (500 msec) from -60 to 50 mV (hp=-60 mV). Activation and inactivation curves were obtained by fitting experimental points with the Boltzmann equation. Maximal conductance (g_CaLmax_) was calculated by interpolating the linear part of the IV curve with a straight line.

Western Blot experiments

Protein lysates were collected from pCMs after 48 hrs incubation with FGF23 1 ng/ml (n=3 differentiation) or vehicle (n=3 differentiation). All procedures were performed on ice or at 4°C. Cells were lysed by scraping in ice-cold RIPA buffer freshly supplemented with protease inhibitors (1:10 dilution) and phosphatase inhibitors (1:50 dilution). Lysates were transferred to 1.5 mL microcentrifuge tubes and centrifuged at 12,000 × g for 15 min at 4°C. The resulting supernatants, containing soluble proteins, were collected and stored at −80°C until further analysis.

For Western blot analysis, we assessed the total ERK and phosphoERK by loading 50 μg of total protein of each sample in two separate gels. Total protein were separated by SDS–PAGE and transferred onto PVDF membranes. Membranes were incubated with the primary antibodies listed below, followed by the appropriate secondary antibodies. Chemiluminescent signal detection was performed using SuperSignal™ West Femto Chemiluminescent Substrate (Thermo Fisher Scientific) and acquired with a ChemiDoc Imaging System (Bio-Rad).

To reprobe the membranes, stripping was performed between Caveolin-1 and ERK detection. Membranes were incubated for 20 min at room temperature in stripping buffer (pH 2.2) containing 1.5% glycine, 0.1% SDS, and 1% Tween-20, followed by extensive washing before subsequent antibody incubation.

Densitometric analysis was performed using Fiji software (NIH). Band intensities were quantified and normalized to Caveolin-1 (Cav-1), used as the loading control. ERK activation was assessed by calculating the ratio of phosphorylated ERK (pERK/Cav1) to total (ERK/Cav1) signal. Normalized values were used for subsequent statistical analyses.

Primary antibodies: anti-caveolin-1 (rabbit, Abcam 1:1000; anti-ERK p42-44 (rabbit, Cell Signaling) 1:1000; anti-Phospho ERK p42-44 (rabbit, Cell Signaling) 1:1000. Secondary antibody: anti-rabbit HRP (1:5000)

**Fig. S1.**

Representative co-staining of SAN cells **(A)** and pCMs **(B)** with anti-HCN4 (yellow) and anti-αKlotho (ab1, SAB3500604 magenta) antibodies with (bottom) and without (top) the specific blocking peptide for the anti-αKlotho antibody. **(C)** Top: representative co-staining of pCM with FGFR1(yellow) and anti-αKlotho (ab2, AF1819, magenta). Bottom: Negative controls. The goat IgG isotype antibody was used as negative control for ab2. Nuclei were stained Hoechst (blue). Scale bar 10µm

**Fig. S2 FGF23 does not affect AP rate and If of SAN cells after short-term exposure**

**(A)**. Mean (black circle) ±SEM and single AP rate values obtained before (open circles) and at the end (open squares) of FGF23 10ng/mL SAN cells perfusion for 74-120 sec. Control: 8.23±0.54 Hz; FGF23: 8.08±0.68 Hz; n=5/3mice, p>0.05 Pair-sample t-Test. **(B)** Left: Sample If traces recorded at -75/-125 mV (hp= -30mV) in the absence and in the presence of FGF23 10ng/mL as indicated. Right: Mean ± SEM of normalized current amplitudes at -75 and -125 mV (n=4/2mice, p>0.05, One sample t-test) **(C)** Mean (black circle) ±SEM and single AP rate values obtained after 30-60 min incubation of SAN cells with either vehicle (open circles) or FGF23 10ng/mL (open squares). Control: 8.45±0.75 Hz, n=9/6 mice; FGF23: 8.71±0.61 Hz; n=8/6mice (p>0.05 sample t-Test). **(D)** Mean (filled circle) ± SEM and single gf values. (**E)** mean If activation curves obtained after 30-60 min incubation of SAN cells with either vehicle (open circles) or FGF23 10ng/mL (open squares).

**Fig. S3. Heterogeneity of mouse SAN Action Potentials.**

Overlays of individual AP waveforms (n=8) obtained after 48 hrs SAN incubation with vehicle.

**Fig. S4**. (A) RT-PCR analysis of α-Klotho (Kl) and FGFR1-4 (R1-4) mRNAs in two different lines of pCMs. Negative control (no cDNA) is also shown. RT-PCR analysis amplified transcripts of all members of the FGFR family and the co-receptor α-Klotho . For α-Klotho, in addition to the expected band (163 bp), we identified one/two additional longer transcripts that may represent alternative non-functional klotho transcripts^6^

**Fig. S5**. Western blot analysis for total ERK and phospho ERK (P-ERK) expression in pCMs treated for 48 hrs with vehicle (control, 3 samples S4, S5, S6) or FGF23 1 ng/ml (3 samples S1, S2 and S3). caveolin 1 (CAV1) has been used as reference. (**B**) Plot of intensity blot analysis for ERK tot, P-ERK and P-ERK/ERKtot ratio, normalized to CAV1. FGF23 induced a significant increase in the P-ERK/ERK tot ratio (*, p<0.05 Student T-test).

References

1.	Yavari A, Bellahcene M, Bucchi A, et al. Mammalian γ2 AMPK regulates intrinsic heart rate. *Nature communications.* 2017;8(1).

2.	Giannetti F, Benzoni P, Campostrini G, et al. A detailed characterization of the hyperpolarization-activated "funny" current (If) in human-induced pluripotent stem cell (iPSC)-derived cardiomyocytes with pacemaker activity. *Pflugers Archiv : European journal of physiology.* 2021;473(7):1009–1021.

3.	Rickert C, Proenza C. ParamAP: Standardized Parameterization of Sinoatrial Node Myocyte Action Potentials. *Biophysical Journal.* 2017;113(4):765–769.

4.	Bucchi A, Baruscotti M, Robinson RB, DiFrancesco D. Modulation of rate by autonomic agonists in SAN cells involves changes in diastolic depolarization and the pacemaker current. *Journal of Molecular and Cellular Cardiology.* 2007;43(1):39–48.

5.	Piantoni C, Paina M, Molla D, et al. Chinese natural compound decreases pacemaking of rabbit cardiac sinoatrial cells by targeting second messenger regulation of f-channels. *eLife.* 2022;11.

6.	Mencke R, Harms G, Moser J, et al. Human alternative Klotho mRNA is a nonsense-mediated mRNA decay target inefficiently spliced in renal disease. *JCI Insight.* 2017;2(20).
